# Comprehensive profiling of alternative splicing and immune landscapes in rectal cancer: implications for mRNA vaccine design and immune subtype stratification

**DOI:** 10.3389/fonc.2026.1780631

**Published:** 2026-04-13

**Authors:** Jiawen Weng, Huangjin Luo, Tong Gao, Yichi Zhang, Yingting Zhuang, Yongmei Dai

**Affiliations:** 1 Departments of Oncology, Shengli Clinical Medical College of Fujian Medical University & Fuzhou University Affiliated Provincial Hospital, Fuzhou, China; 2 Department of Gynecology, Shengli Clinical Medical College of Fujian Medical University& Fuzhou University Affiliated Provincial Hospital, Fuzhou, China; 3 School of Basic Medical Sciences, Fujian Medical University, Fuzhou, China; 4 School of Pharmacy, Fujian Medical University, Fuzhou, China

**Keywords:** *FAM135A*, immune subtypes, mRNA vaccines, rectal cancer, tumor antigen

## Abstract

**Background:**

mRNA vaccines have emerged as a promising platform for cancer immunotherapy, particularly following the success of COVID-19 vaccines. However, the development of cancer vaccines presents challenges such as difficulties in antigen prediction and poor immunogenicity, especially in identifying and delivering highly immunogenic tumor-specific antigens. The variability and low immunogenicity of tumor antigens further complicates this process.

**Methods:**

This study utilized public data and bioinformatics analysis to identify potential tumor antigens in cancer and characterize different immune subtypes. This approach aims to guide the development of cancer mRNA vaccines with enhanced immune response.

**Results:**

In the cancer genome atlas rectal adenocarcinoma(TCGA-READ), Exon skipping was the most common alternative splicing event in TCGA-READ, whereas mutually exclusive exons were the least common. We identified 4480 upregulated and 3328 downregulated AS events, with missense mutations being the most frequent. A total of 217 potential antigen genes were identified by intersecting upregulated AS anomalies and frameshift mutations. *FAM135A, GAR1*, and *CDIPT* have been highlighted as potential antigens, with *FAM135A* showing significant correlation with immune cell infiltration. TCGA-READ samples stratified into C1 and C2 subtypes by survival and immune profiles revealed that C2 tumors derive markedly greater benefit from CTLA-4/PD-1 blockade than C1 tumors, underscoring the value of molecular subtyping in guiding precision immunotherapy.

**Conclusions:**

*FAM135A* was identified as a potential tumor vaccine antigen, and an immune subtype analysis was conducted in different patients. This study provides guidance for the development of novel cancer mRNA vaccines.

## Highlights

- Exon skipping dominates alternative splicing events in rectal cancer, while mutually exclusive exons are rare.
- Integration of upregulated AS anomalies and frameshift mutations identifies 217 potential tumor antigen genes, with FAM135A emerging as a key candidate strongly correlated with immune cell infiltration.
- Rectal cancer is stratified into two immune subtypes (C1/C2) with distinct survival patterns; subtype C2 shows enhanced responsiveness to CTLA-4 inhibition.
- Findings provide novel targets for mRNA vaccine design (e.g., FAM135A) and a framework for immune-driven patient stratification in rectal cancer.

## Introduction

Rectal cancer, which accounts a substantial proportion of colorectal malignancies, is a significant global health burden. Despite these improvements and the development of novel therapeutic approaches including minimally invasive surgical techniques (1, 2), neoadjuvant chemoradiotherapy (3–5), and targeted therapies, the prognosis of advanced rectal cancer remains challenging. Metastatic rectal cancer (mRC) continues to pose significant therapeutic challenges, with 5-year survival rates remaining relatively low at approximately 13-15% (6). Given the distinct biological characteristics and clinical challenges of rectal cancer, there is an urgent need for continued research and development of innovative treatment strategies tailored specifically to this disease. Precision medicine approaches, immunotherapy combinations, and novel targeted agents offer promising avenues for improving outcomes in patients with rectal cancer, particularly those with advanced or metastatic disease who exhibit a limited response to conventional immune checkpoint blockade (ICB) (7, 8). Concurrently, integrated pharmacology approaches and network-based strategies have increasingly been applied to decipher the complex mechanisms of tumor progression and to screen for effective therapeutic agents or targets, providing a complementary perspective to vaccine development (9–12).

Tumor vaccines represent a frontier in active immunotherapy, with mRNA-based platforms gaining prominence owing to their transient expression kinetics and avoidance of insertional mutagenesis risks (13). Clinical trials have been initiated for various malignancies, including non-small cell lung cancer, glioblastoma, pancreatic cancer, and prostate cancer (14, 15), and combining mRNA vaccines with immune checkpoint blockade (ICB) may synergistically enhance anti-tumor immunity (16). However, key obstacles persist: identifying immunogenic tumor-specific antigens (TSAs)​​ and overcoming the immunosuppressive tumor microenvironment (TME), both exacerbated in rectal cancer by its inherently immunologically “cold” phenotype (17). Recent studies have revealed that ​disrupted nonsense-mediated decay (NMD) permits aberrant transcripts, containing frameshift mutations or abnormal splicing patterns, to generate immunogenic neoantigens (18, 19). This positions abnormal splicing events as dual-purpose biomarkers: sources of TSAs for vaccines and predictors of responses to ICB.

**Figure 1:**
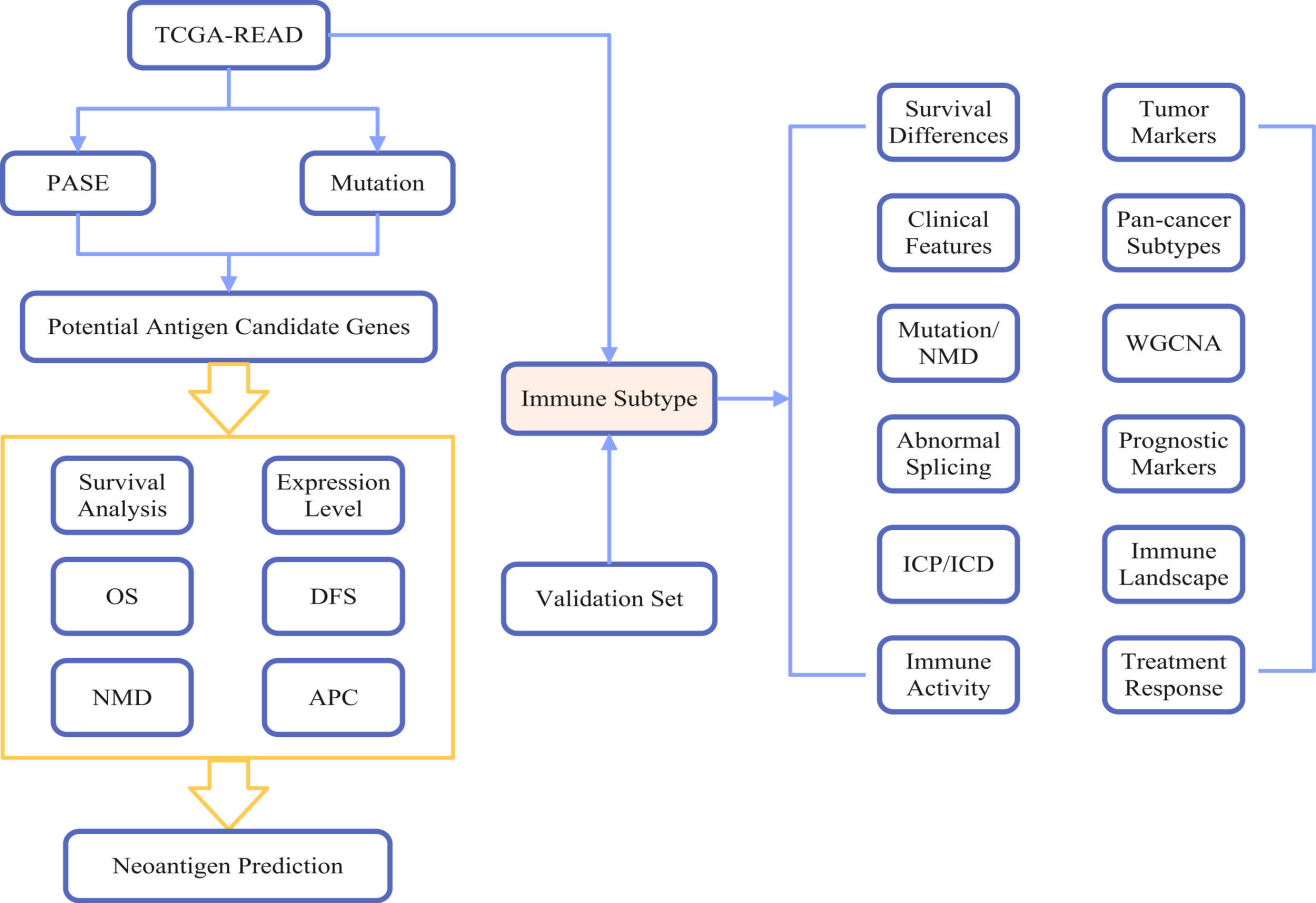
Comprehensive workflow of the study. Abbreviations: TCGA-READ, The Cancer Genome Atlas Rectal Adenocarcinoma; PASE, Potential Alternative Splicing Events; OS, Overall Survival; DFS, Disease-Free Survival; NMD, Nonsense-mediated mRNA Decay; APC, Antigen Presenting Cells; ICP, Immune Checkpoint; ICD, Immunogenic Cell Death; WGCNA, Weighted Gene Co-expression Network Analysis.

Here, we employed an integrated bioinformatics approach​ to:1)Identify NMD-related neoantigens​ in rectal cancer by screening aberrant splicing events and frameshift mutations and 2)characterize immune subtypes​ to stratify patients for vaccine responsiveness. Our comprehensive workflow (detailed in **Figure 1**) leveraged public multiomics data to guide mRNA vaccine development for rectal cancer.

## Materials and methods

### Data sources and preprocessing

Data Sources: Splicing analysis data for rectal cancer (READ) were downloaded from TCGA Splice-Seq database (https://bioinformatics.mdanderson.org/TCGASpliceSeq/singlegene.jsp). Samples were screened when the percentage splicing index (PSI) value was not 0 or 1 to ensure data quality and the amount of valid information. Given the non-normal distribution of PSI values, the Wilcoxon rank-sum test was primarily used. Key findings were further verified using Welch’s t-test to ensure robustness against variance heterogeneity. Gene expression profiles, mutation data, and clinical and survival information for TCGA-READ were downloaded from the Xena GDC Hub (https://xenabrowser.net/datapages/) and integrated with survival data from the cBioPortal database (http://www.cbioportal.org/) to ensure the completeness of sample information. The GSE87211 dataset (20) was obtained from the GEO database (https://www.ncbi.nlm.nih.gov/geo/query/acc.cgi?acc=GSE87211), including expression profiles and clinical information of samples, for external validation analysis. All data analyses were based on de-identified publicly available data in compliance with ethical guidelines, such as those of the REDCap.

The data preprocessing process included the following steps: Map probes to genes based on the annotation file and remove probes that cannot be mapped. For multiple probes corresponding to the same gene, the one with the highest expression value was selected as the expression level of that gene. The data for all samples were standardized to ensure comparability between different data sources.

### Data analysis

#### Abnormal splicing event analysis

Statistical testing of PSI values using the Wilcoxon test was performed to screen for significant splicing events between tumor and normal samples, with a significance threshold set at *P* < 0.05. Given the non-normal distribution of PSI values, the Wilcoxon rank-sum test was primarily used. Key findings were further verified using Welch’s t-test to ensure robustness against variance heterogeneity. Splicing events were classified by type into exon skipping (ES), alternative splicing sites (ASS), intron retention (IR), and mutually exclusive exons (ME). Significantly different splicing events were visualized using heatmaps and volcano plots to display events that were significantly upregulated or downregulated in tumor samples.

#### Differential gene expression analysis

The standardized TCGA-READ expression profiles were analyzed using the limma package in R. Screened genes were identified based on the differential expression analysis results, setting the threshold as |LogFC| > 0.58(|FC|>1.5) and *P* < 0.01. Volcano plots and heat maps were then generated to intuitively display the distribution patterns of differentially expressed genes in tumor and normal samples.

#### New antigen prediction

Intersecting differentially expressed abnormal splicing genes, and frameshift mutation genes were used to preliminarily screen potential antigen candidate genes. Using The Cancer Immunome Atlas (TCIA) database (https://tcia.at/), we predicted the binding ability of antigen peptides encoded by candidate genes to HLA molecules to identify tumor neoantigens with high immunogenicity. The antigen peptide sequences were analyzed, the HLA binding strength of each candidate gene was assessed, and an antigen prediction result table was generated.

#### Immune cell infiltration analysis

Immune infiltration score data were downloaded from the TIMER2 database (https://compbio.cn/timer3/) and immune cell infiltration information was extracted from rectal cancer samples. Spearman’s correlation analysis was used to assess the relationship between the expression levels of candidate antigen genes and the proportion of different immune cells. The CIBERSORT algorithm was applied to the GSE87211 dataset to calculate immune cell infiltration scores and validate the reliability of TCGA analysis results.

#### Immune cell analysis and immune landscape visualization

The R package was used to sequentially evaluate immune, stromal, and tumor purity for different TCGA-READ subtypes using the mean expression levels of GZMA and PRF1 as CYT scores. Next, the immune infiltration score file from the TIMER2 database was downloaded, the data related to TCGA-READ samples were screened, and the CIBERSORT score data were used for further analysis. CIBERSORT was used to calculate the immune infiltration score and perform the Wilcoxon test for each immune score indicator to determine statistical differences.

We downloaded data on 28 pan-cancer immune cell subpopulations from the literature (21) and analyzed the enrichment levels of each immune cell subpopulation in TCGA-READ using the ssGSEA algorithm. Next, we utilized the monocle package to perform DDRTree dimensionality reduction on TCGA-READ, followed by a pseudo-time series analysis. We extracted the first two principal components and calculated their correlations with the 28 pan-cancer immune cell subpopulations. Based on the pseudo-time-series, distribution of TCGA-READ samples, we further subdivided the subtypes. One-way analysis of variance (ANOVA) was used to statistically analyze the enrichment of the 28 pan-cancer immune cell subpopulations in each subtype.

To ensure the robustness and algorithm-independence of our immune subtyping, we performed orthogonal validation using Non-negative Matrix Factorization (NMF). The NMF algorithm was executed to evaluate the stability of the stratification, and Principal Component Analysis (PCA) was subsequently used to visually confirm the separation of the resulting clusters.

#### Consensus clustering analysis

Immune-related gene sets were downloaded from the ImmPort database (https://www.immport.org/shared/home) and immune genes associated with rectal cancer prognosis were screened. The ConsensusClusterPlus package was used to perform consensus clustering on immune-related genes to determine the optimal number of clusters (k-value). Based on the clustering results, immune subtypes were defined and differences in clinical and molecular features between different subtypes were analyzed.

#### Weighted gene co-expression network analysis network construction

We screened the expression levels of differentially expressed genes in TCGA-READ and used the WGCNA package to construct a co-expression network for the TCGA-READ expression matrix. First, we calculated the soft threshold (softpower) from 1 to 10, scaling fit index and average connectivity of the network with respect to the soft power parameter. The soft power was set to 4 when the scaling fit index was > 0.8 to ensure a scale-free network. Finally, the co-expression network was constructed and the prognostic modules within it were identified, ensuring that each module contained a minimum of 30 genes and merging modules with a correlation greater than 0.7. The Pearson correlation between genes in the modules and module feature genes was calculated, and hub genes were screened using the criteria |cor| > 0.9 and *P < 0.001*.* A* risk score was constructed based on the multifactorial Cox model of these hub genes.

#### Survival analysis and functional enrichment

Univariate Cox regression analysis was used to identify genes associated with prognosis and calculate their association with overall survival (OS) and disease-free survival (DFS). GO biological process, molecular function, cellular component, and KEGG pathway functional enrichment analyses were performed for the selected genes to reveal their potential biological functions. Kaplan-Meier analysis of survival differences between different immune subtypes and survival curve plots.

## Results

### Analysis of abnormal splicing events

**Figure 2:**
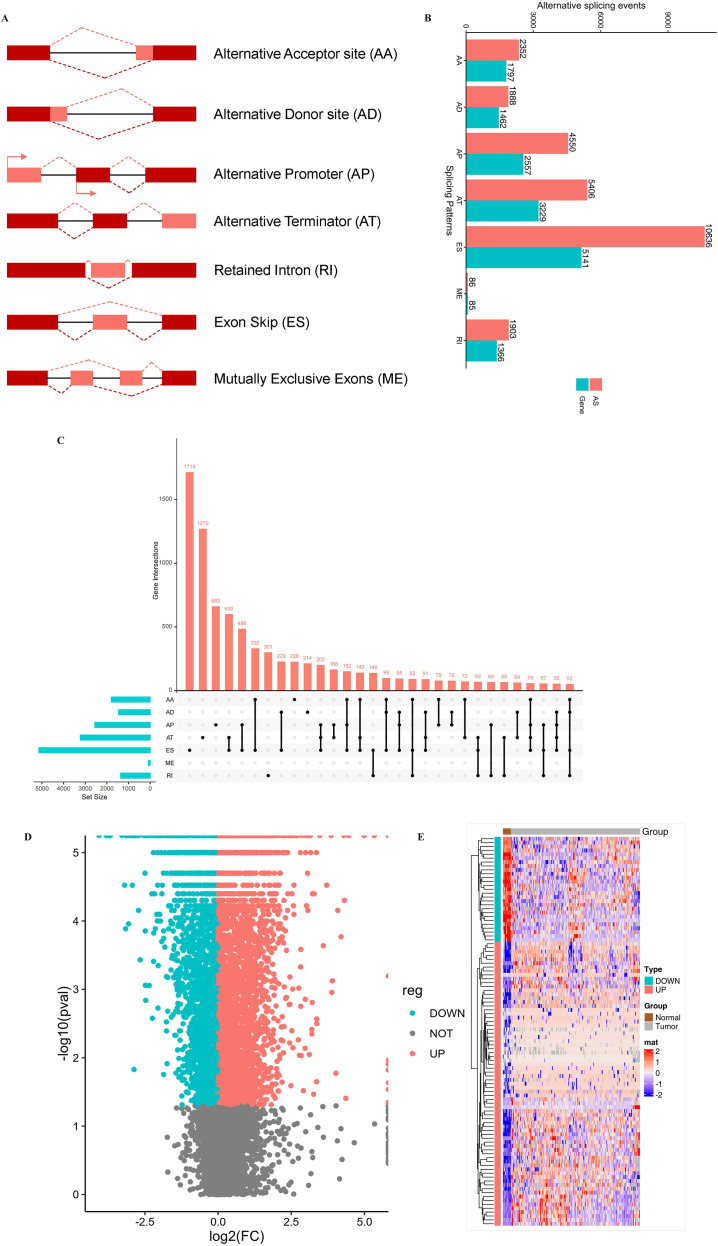
Overview of variable splicing events in READ. **(A)** Schematic diagram of seven types of variable splicing events. **(B)** Statistics on the number of occurrences and the number of genes involved in variable splicing events. **(C)** UpSetR plot of genes involved in different variable splicing events. **(D)** Volcano plot of differential analysis of variable splicing events. **(E)** Heat map of the top 100 differentially expressed variable splicing events.

Seven types of abnormal splicing events were detected (**Figure 2A**), including exon skipping (ES), alternative 5’ or 3’ splicing sites (A5SS and A3SS), intron retention (IR), and mutually exclusive exons (ME). Among these, exon skipping (ES) was the most common splicing type, dominating both the number of occurrences and the number of genes involved. A total of 4,480 upregulated and 3,328 downregulated AS events were detected in tumor samples (**Figure 2**), indicating significant changes in abnormal splicing patterns in tumor tissues. A PSI heat map of the top 100 AS events is shown in **Figure 2**. Detailed lists of differentially expressed AS events are provided in **Table 1**. The distribution and significance of these events form the basis of subsequent antigen screening. Given the non-normal distribution of PSI values, the Wilcoxon rank-sum test was primarily used. Key findings were further verified using Welch’s t-test to ensure robustness against variance heterogeneity (**Supplementary Table 2**).

**Table 1:** TCIA for prediction of novel antigens.

Patient barcode	Disease	Gene	Peptide	HLA-alleles
TCGA-AG-4021	CRC	FAM135A	ATYASSTF	HLA- A*02:01
TCGA-AG-4021	CRC	FAM135A	ISATYASSTF	HLA- A*02:01

### Screening of differentially expressed genes and potential antigens

Through intersection analysis of differentially expressed genes, abnormal splicing event genes, and frameshift mutation genes, 217 potential antigen candidate were identified (**Supplementary Figure 1**). These genes were identified through GO functional analysis and were found to be mainly involved in immune-related biological processes, such as antigen processing and presentation and immune signaling. (**Supplementary Figure 1B**).

**Figure 3:**
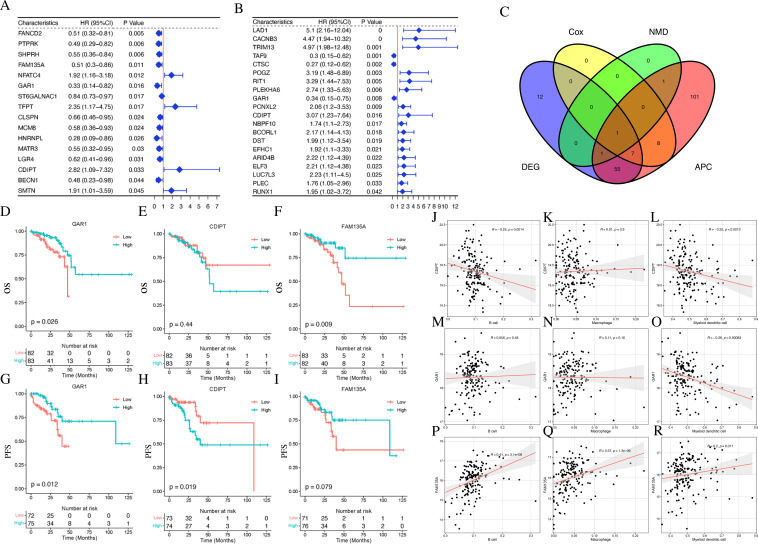
Survival analysis of potential antigen candidate genes. **(A, B)** Univariate cox analysis of OS and DFS in TCGA-READ. **(C)** Venn diagram of the intersection of potential antigen candidate genes. **(D–F)** Curves for prognostically relevant potential antigen candidate genes in OS. **(G–I)** Curves for prognostically relevant potential antigen candidate genes in DFS. **(J–R)** Correlation between potential antigen candidate genes and immune cell infiltration. DFS, disease-free survival; OS, overall survival.

We conducted a comprehensive screening for prognostic and immune-related genes as potential antigenic genes using the TCGA-READ database. We initiated survival analysis and performing individual Cox analyses (P < 0.05) to identify the genes associated with overall survival (OS) and disease-free survival (DFS). This analysis revealed 16 genes linked to OS and 20 genes linked to DFS (**Figure 3A**). The intersection of these two gene sets identified *GAR1* and *CDIPT*, which were further evaluated for their associations with survival outcomes. Subsequent Kaplan-Meier survival analysis with log-rank testing confirmed that *GAR1* expression exhibited consistent correlations with both disease-free survival (DFS) and overall survival (OS): low *GAR1* expression was significantly associated with poorer outcomes in both cohorts (**Figure 3D**; p=0.026 and 0.012, respectively). For *FAM135A*, a significant OS association was observed exclusively in the first cohort (low vs. high expression: *P = 0.009*; **Figure 3**), whereas its correlation with DFS and CDIPT’s survival associations were not uniformly significant across cohorts (**Figures 3E, H**).

To explore the relationship between the expression of these potential antigen candidate genes and immune cell distribution, immune infiltration scores was used. Our findings indicate that *FAM135A* expression was positively correlated with B cell, macrophage, and myeloid dendritic cell infiltration, whereas *CDIPT* was negatively correlated with B cell and myeloid dendritic cell infiltration. *GAR1* was negatively correlated with myeloid dendritic cell infiltration(**Figure 3J**).

Subsequently, we identified *FAM135A* as a potential antigenic gene through the intersection of our findings, as shown in the Venn diagram (**Figure 3**). Further investigation of the TCIA database suggested that *FAM135A* could be a novel antigen (**Table 1**).

### Immune subtype classification

**Figure 4:**
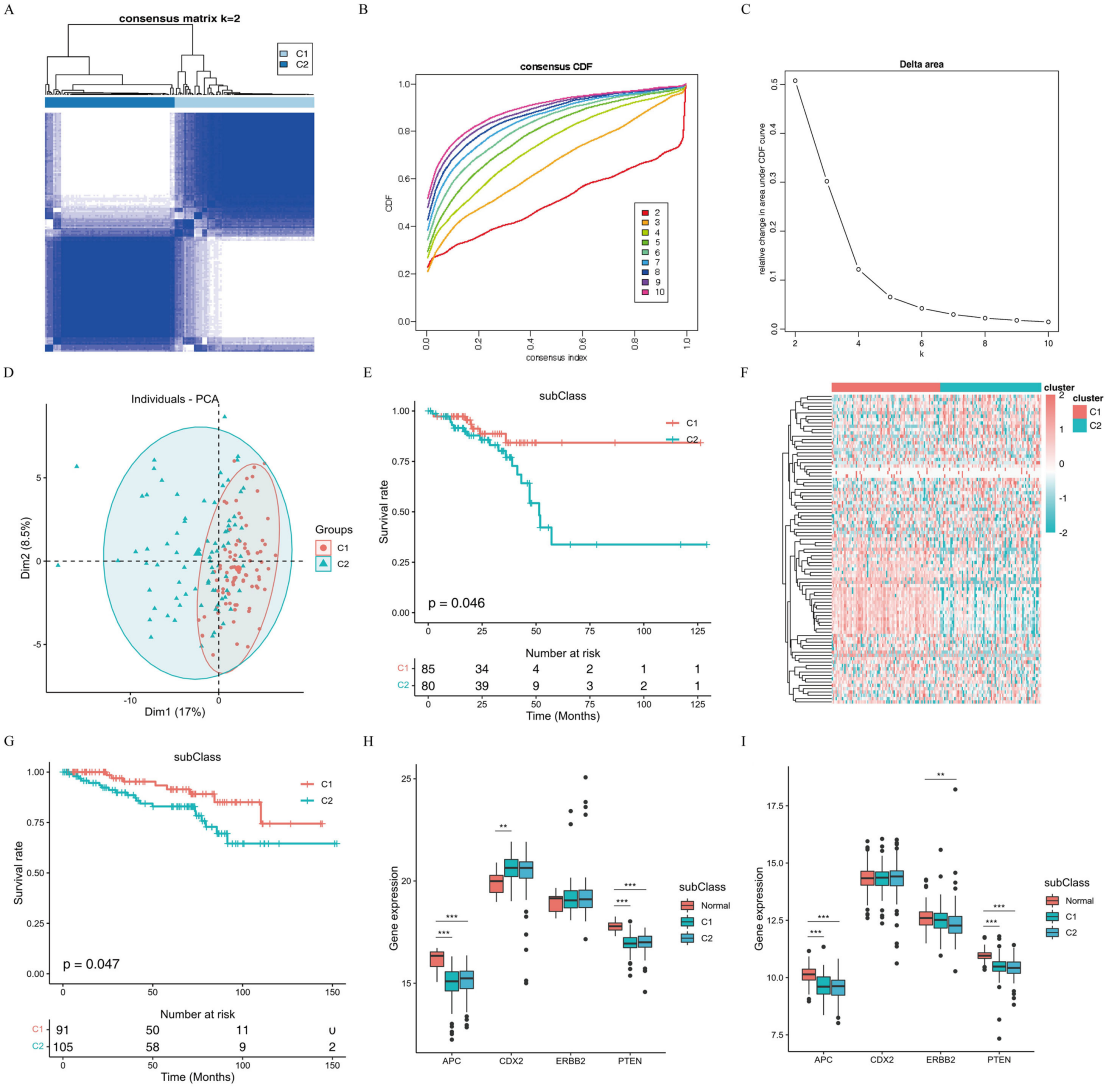
Identification of immune subtypes. **(A)** Clustering plot of prognostically relevant immune genes in TCGA-READ. **(B)** Cumulative distribution function (CDF) graph. **(C)** The relative change trend of the area under the CDF Curv. **(D, E)** PCA analysis and survival analysis of TCGA-READ patients. **(F)** Heatmap of prognostic immune-related gene expression for two subtypes of READ in TCGA-READ. **(G)** Survival analysis of cancer patients in the GEO dataset(GSE87211). **(H, I)** Differential analysis of biomarkers among different subtypes in TCGA-READ and GEO dataset(GSE87211).

COX univariate analysis was used to assess the correlation between immune factors and prognosis: 93 genes were found to be associated with prognosis. Subsequent cluster analysis revealed two subtypes in TCGA-READ, and significant survival differences were observed between these subtypes (**Figure 4A**). To validate the prognostic value of this immune stratification in an independent cohort, we performed survival analysis on the GSE87211 dataset. The results similarly demonstrated that patients in the two subtypes exhibited significantly different clinical outcomes (**Figure 4**), consistent with the findings in the TCGA cohort. To further confirm the robustness of the C1/C2 stratification and rule out algorithm-specific biases, we utilized Non-negative Matrix Factorization (NMF) as an alternative clustering approach. The NMF consensus matrix at k = 2 exhibited distinct boundaries and high intra-cluster consistency (**Supplementary Figure 2**), yielding highly concordant cluster assignments (C1 = 109, C2 = 57) compared to the original consensus clustering. Additionally, PCA based on NMF classifications demonstrated clear spatial separation between the two subtypes (**Supplementary Figure 2**). These validation metrics collectively instill high confidence in the stability and biological relevance of our C1/C2 immune stratification.

Adenomatous Polyposis Coli(*APC*), *CDX2, ERBB2* and *PTEN* are common diagnostic and prognostic indicators for colorectal cancer. We investigated the relationship between different subtypes and these markers. In TCGA-READ data, the expression levels of *APC, CDX2* and *PTEN* differed between tumor and normal tissues but showed no variance among different subtypes. Conversely, in the GSE87211 dataset, the expression levels of *APC, ERBB2* and *PTEN* were different between tumor and normal tissues, with no variations observed among the different subtypes (**Figure 4H**). A comparison of clinical characteristics between subtypes revealed statistically significant differences in the distribution of pathologic lymph node metastasis (pathologic N) between the two subtypes (*P* = 0.026, Fisher’s exact test). Specifically, the proportion of N0 was as high as 70.5% in subtype 1 but only 26.5% in subtype 2; and the N1/N2 metastasis ratio was significantly higher in subtype 2 (29.5% in subtype 1 and 73.5% in subtype 2). In contrast, there were no statistically significant differences in the distribution of pathological T stage (*P* = 0.201) or distant metastasis (pathologic M, *P* = 0.387) between the two subtypes (**Supplementary Figure 3**).

### Analysis of immune activity in two subtypes

**Figure 5:**
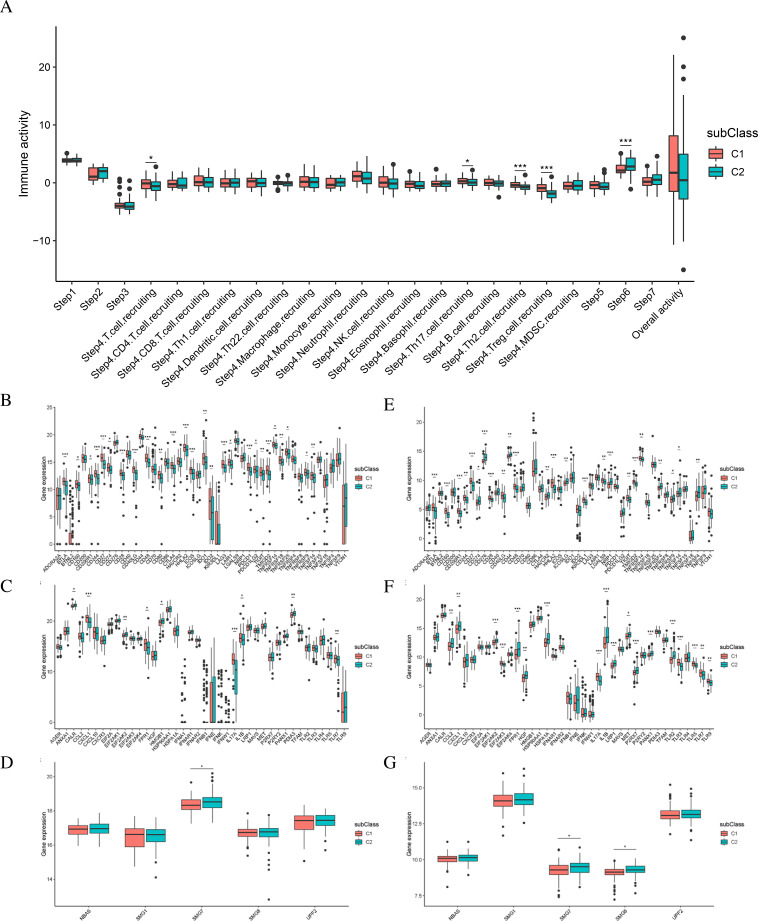
The relationship between immune subtypes and immunogenic cell death regulators as well as NMD Factors. **(A)** Immune activity analysis of TCGA-READ subtypes. **(B-D)** Differential expression of immune checkpoints, immunogenic cell death regulators, and NMD factors among immune subtypes in TCGA-READ. **(E-G).** Differential expression of immune checkpoints, immunogenic cell death regulators, and NMD factors among immune subtypes in GEO database (GSE87211).

Immune activity-related data from TCGA-READ were obtained from the TIP database. Differential analysis of immune activity scores was conducted between different subtypes, revealing significant differences in certain immune activity scores between the two subtypes (*P* < 0.05) (**Figure 5**).

We obtained a list of immune checkpoint (ICP) genes from the literature (22, 23), which together comprise 47 ICP genes. Furthermore, we assembled a list of 43 immunogenic cell death (ICD) regulatory genes from a previous research (22–26). Differences in the expression of these genes were examined in the two subtypes of TCGA-READ, revealing 26 differential ICP genes, nine ICD genes, and one NMD factor (**Figure 5B**). Analysis of the GSE87211 dataset revealed 24 differential ICP genes, 18 ICD genes, and two NMD factors (**Figure 5E**).

**Figure 6:**
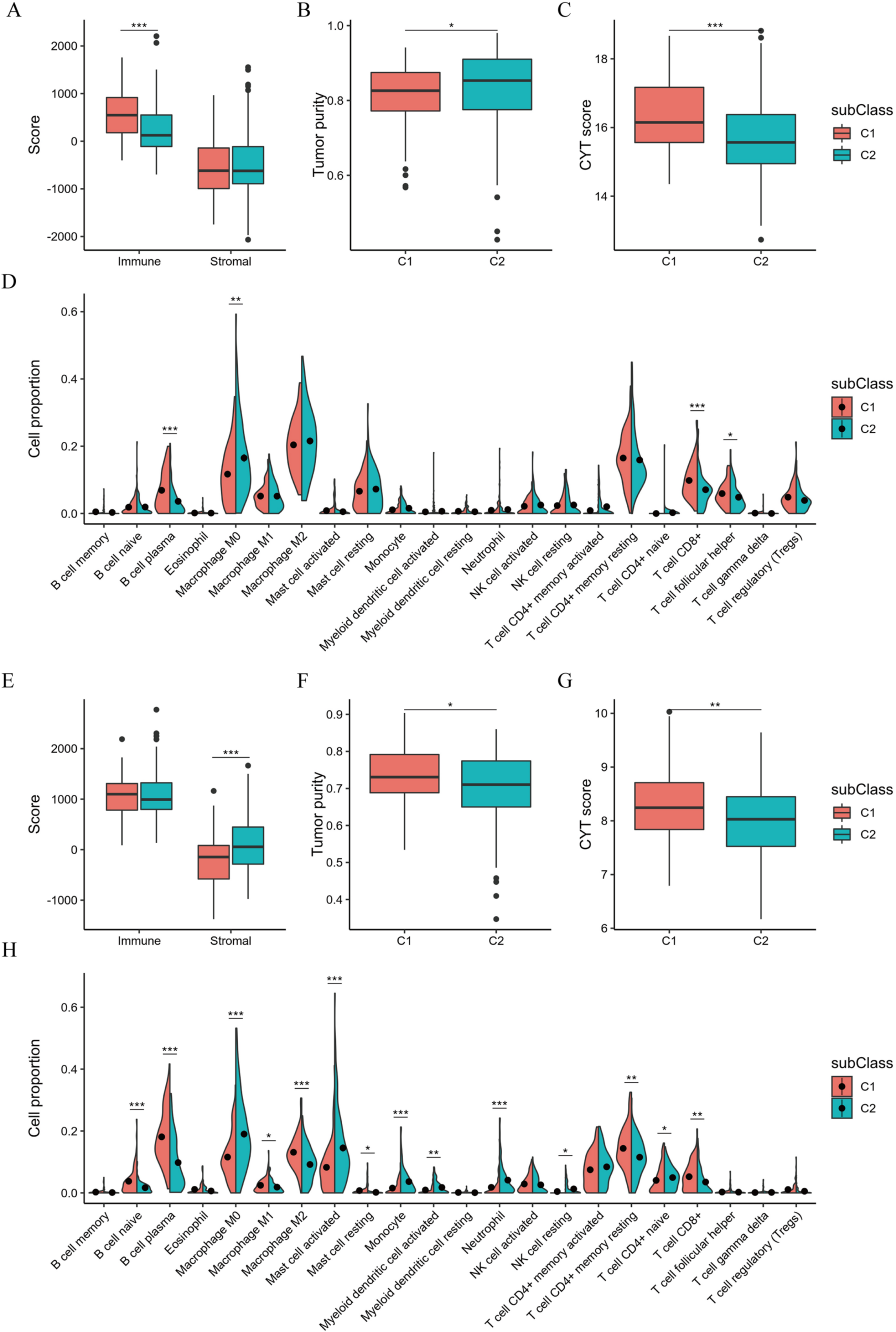
The relationship between immune cell characteristics and immune subtypes. **(A-D)** Differences in immune scores, stromal scores, tumor purity, CYT Scores, and proportions of immune cells among TCGA-READ subtypes. **(E-H)**. Differences in immune scores, stromal scores, tumor purity, CYT scores, and proportions of immune cells among GEO database (GSE87211) subtypes.

The immune infiltration scoring files for TCGA-READ were obtained from the TIMER2 database. For the GSE87211 data, CIBERSORT was used to calculate the immune infiltration scores. In the TCGA-READ dataset, significant differences were observed in immune scores, tumor purity, and CYT scores among different subtypes, and the distribution proportions of the four immune cell types differed (**Figure 6A**). In the GSE87211 dataset, there were significant differences in stromal scores, tumor purity, and CYT scores between the two subtypes, and the distribution proportions of the 14 immune cell types differed (**Figure 6E**).

### Characterization of the immune landscape

**Figure 7:**
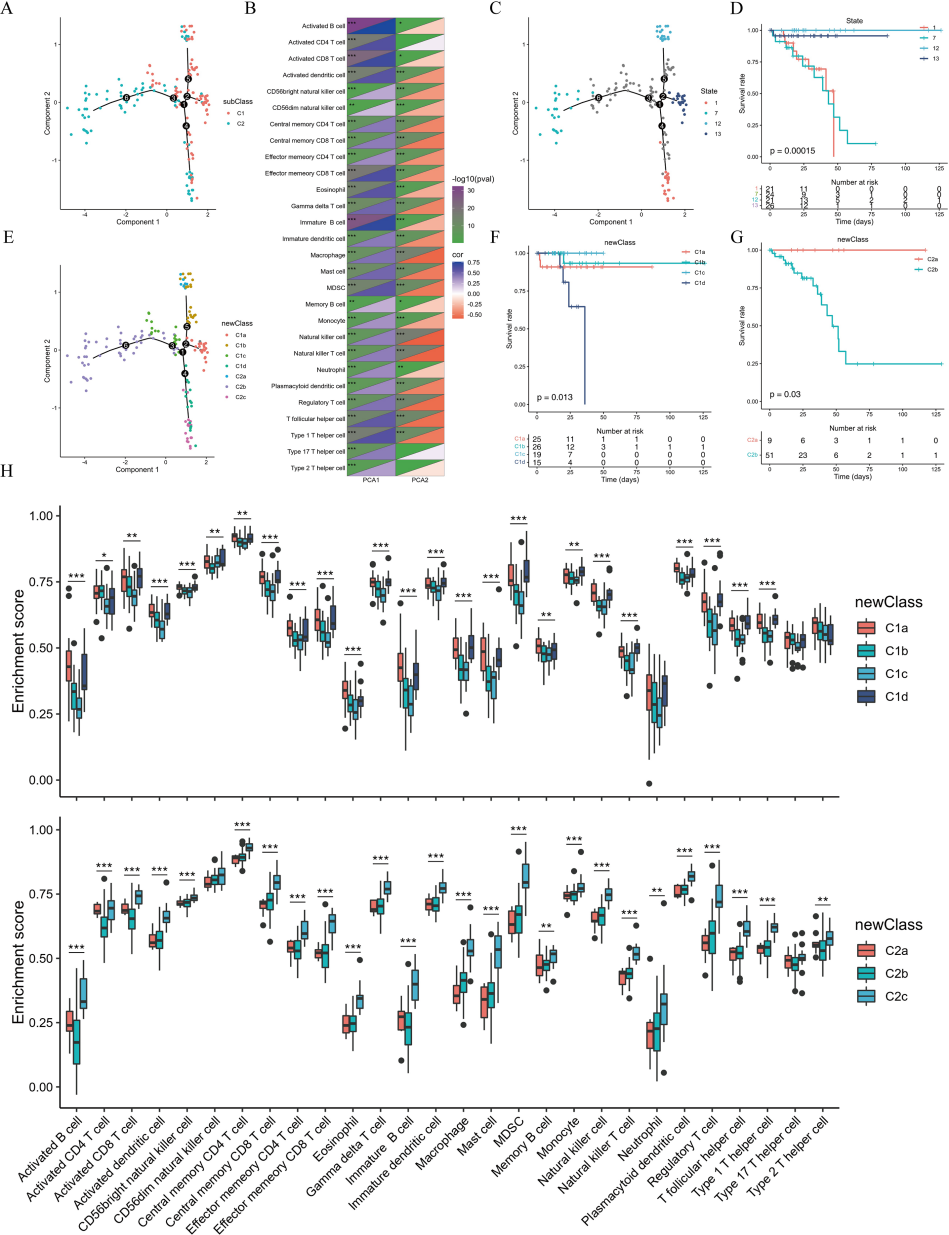
Characterization of the immune landscape in TCGA-READ. **(A)** Pseudotime trajectory analysis and spatial distribution of different subtypes following DDRTree dimensionality reduction in the TCGA-READ cohort. **(B)** Correlations between the principal components (PC1/PC2) and various immune cell subpopulations. **(C, D)** Distribution and Kaplan-Meier survival analysis of samples located at the extreme positions of the spatial trajectory. **(E)** Subdivision of C1/2 subtypes based on the distribution in PCA. **(F, G)**. Survival analysis among the distinct sub-clusters derived from the C1 and C2 subtypes. **(H)** Differential enrichment analysis of 28 pan-cancer immune cell subpopulations across the sub-clusters of C1 and C2.

To characterize the immune microenvironment, signature gene sets for 28 immune cell subpopulations were obtained from a previous pan-cancer study (21). Single-sample gene set enrichment analysis (ssGSEA) was subsequently employed to quantify the enrichment levels of these immune cell subsets in the TCGA-READ cohort. Initially, the monocle R package was utilized to perform DDRTree dimensionality reduction, followed by pseudotime trajectory analysis on the TCGA-READ samples (**Figure 7**). We then extracted the two primary components (principal components, PCs) from this trajectory to evaluate their correlations with the 28 immune cell subpopulations. The analysis revealed that PC1 (X-axis) exhibited a strong positive correlation with activated B cells, immature B cells, and activated CD8+ T cells, whereas PC2 (Y-axis) was primarily negatively correlated with macrophages, natural killer (NK) cells, and natural killer T (NKT) cells (**Figure 7**).

Based on their spatial distribution in the reduced-dimension space, samples located at the extreme ends of the trajectory were categorized into four distinct groups. Survival analysis demonstrated significant prognostic differences among these four extreme-position groups (**Figure 7C**). Furthermore, according to their spatial positions, the previously defined C1 subtype could be further stratified into four sub-clusters that displayed significant survival disparities. Similarly, the C2 subtype was subdivided into three sub-clusters, with a notable survival difference observed specifically between the C2a and C2b subsets (**Figure 7E**). Finally, Analysis of Variance (ANOVA) was applied to compare the enrichment scores of the 28 pan-cancer immune cell subpopulations across the various sub-clusters of C1 and C2. The results indicated that the majority of these immune subsets exhibited statistically significant differential enrichment across the sub-clusters (**Figure 7**), highlighting distinct immunological profiles within the tumor microenvironment.

### WGCNA employing to screen for prognosis-related hub genes and construction of risk model

WGCNA was employed to construct a co-expression network for the TCGA-READ expression matrix with a soft power set to 4 to ensure scale-free network properties. Ultimately, genes were classified into 10 modules, with the turquoise module containing the most genes and the magenta module the least genes. The expression values of genes in the black, brown, green, gray, pink, red, turquoise, and yellow modules were significantly different among the subtypes (**Supplementary Figure 4A**). Single-factor Cox analysis was conducted on the eigengenes of each module, and modules associated with prognosis were identified (grey, red, and turquoise modules) (**Supplementary Figure 4**). GO enrichment analysis was performed on the genes in the gray, red, and turquoise modules (**Supplementary Figure 4H**).

**Figure 8:**
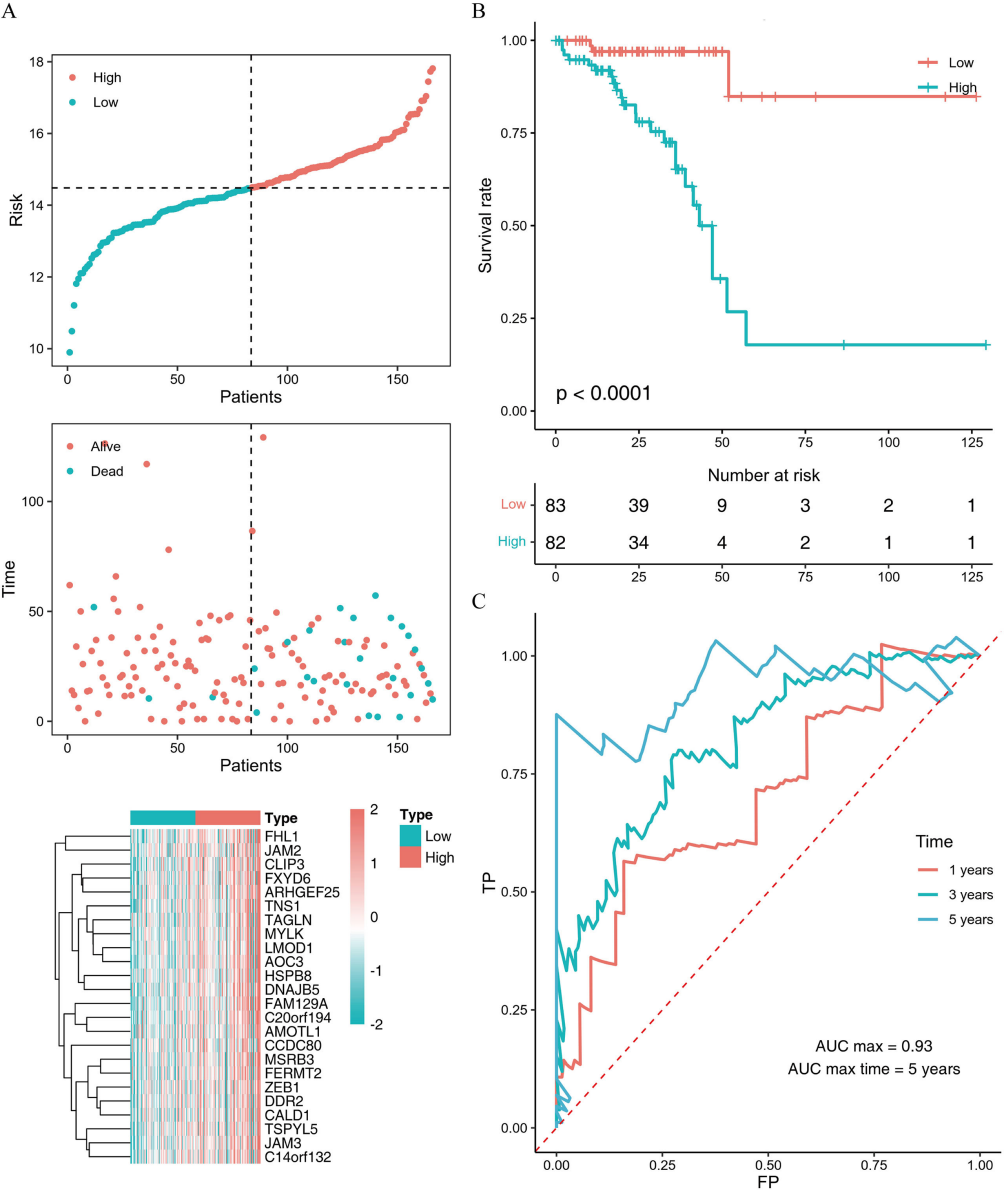
Construction of risk score for hub genes in prognostically relevant modules. **(A)** Survival statistics and expression level correlation analysis of TCGA-READ samples based on risk score. **(B)** Survival analysis of TCGA-READ samples stratified by different risk scores. **(C)** ROC curve of risk score for tumor prognosis.

The correlation between each gene in the prognostic modules and eigengenes was calculated, and hub genes were selected based on |cor| > 0.9 and p < 0.001. A total of 24 hub genes were identified (*FAM129A, FHL1, MYLK, JAM2, HSPB8, LMOD1, DNAJB5, FXYD6, ARHGEF25, C20ORF194, TNS1, MSRB3, CLIP3, ZEB1, AOC3, JAM3, AMOTL1, DDR2, TSPYL5, FERMT2, TAGLN, C14ORF132, CCDC80, CALD1*). A risk score was constructed based on the expression levels of the hub genes. Tumor samples were divided into two groups according to the median score. Survival analysis revealed a significant difference between the two score groups and the ROC curve also demonstrated good diagnostic performance (**Figure 8**).

### Analysis of immune therapy efficacy in different subtypes

**Figure 9:**
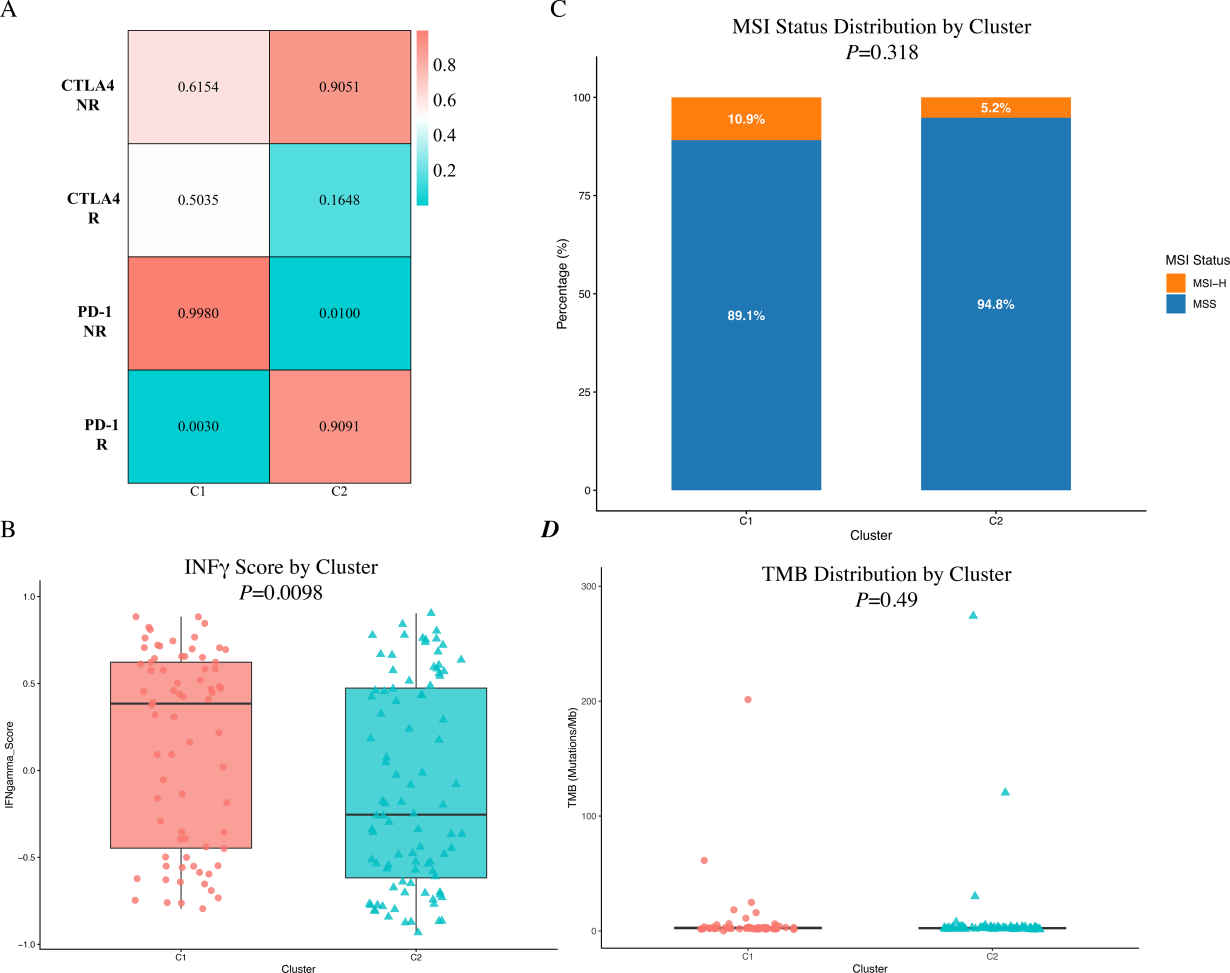
Evaluation of immunotherapy response and immunogenic biomarkers in TCGA-READ subtypes. **(A)** Subclass mapping (SubMap) analysis predicting the potential clinical response to anti-CTLA-4 and anti-PD-1 therapies in the C1 and C2 subtypes. The C2 subtype demonstrates a significantly higher potential response rate to both immune checkpoint inhibitors (nominal P < 0.05). **(B)** Boxplots comparing the Interferon-gamma (IFN-γ) scores between the C1 and C2 subtypes. The IFN-γ score is significantly enriched in subtype C2, indicating a more active, T-cell-inflamed tumor microenvironment. **(C)** Stacked bar charts illustrating the distribution of Microsatellite Instability (MSI) status across the two subtypes. No significant difference was observed in the proportion of MSI-H patients between C1 (10.9%) and C2 (5.2%) (P = 0.318). **(D)** Boxplots comparing the Tumor Mutational Burden (TMB) levels between the C1 and C2 subtypes, showing no statistically significant differences. Abbreviations: R, responders; NR, non-responders; CTLA-4, cytotoxic T-lymphocyte-associated protein 4; PD-1, programmed cell death protein 1; IFN-γ, Interferon-gamma; MSI, microsatellite instability; MSI-H, high microsatellite instability; MSS, microsatellite stable; TMB, tumor mutational burden.

Roh (27)evaluated the therapeutic effects of CTLA-4 and PD-1 inhibitors in 47 melanoma patients and detected changes in gene expression levels before and after inhibitor treatment, categorizing the patients into responders (R) and non-responders (NR). The gene expression profile data of TCGA-READ colorectal cancer patients were analyzed using the GenePattern plugin submap to assess the potential therapeutic effects of CTLA-4 and PD-1 inhibitors. The results showed that the potential response rate of subtype C2 to CTLA-4 and PD-1 inhibitor treatment was significantly higher than that of subtype C1 (nominal *P* < 0.05) (**Figure 9**). These findings suggest that patients with C2 subtype may benefit more from immune checkpoint inhibitor therapy, highlighting the potential value of subtype analysis in guiding precision immunotherapy. Considering the limitations of extrapolating ICB responses from a melanoma cohort, we further validated the immunogenic differences between the subtypes using orthogonal biomarkers. We evaluated the Interferon-gamma (IFN-γ) signature, which is a robust indicator of a T-cell-inflamed microenvironment and clinical response to ICBs. Consistent with the SubMap predictions, the IFN-γ score was significantly enriched in subtype C2 compared to C1 (**Figure 9**), supporting a more active anti-tumor immune state. Furthermore, we assessed the distribution of classical predictive biomarkers, including Microsatellite Instability (MSI) and Tumor Mutational Burden (TMB). Interestingly, no significant differences were observed in MSI status (MSI-H: 10.9% in C1 vs. 5.2% in C2, p = 0.318; **Figure 9**) or TMB levels (**Figure 9**) between the two subtypes.

## Discussion

Despite significant advances in the management of localized rectal cancer (RC), metastatic rectal cancer (mRC) continues to pose a formidable clinical challenge, accounting for the majority of RC-related mortality worldwide (28, 29). Even with multimodal treatment approaches—including neoadjuvant chemoradiotherapy, surgical resection, and systemic therapy—patients with mRC have poor 5-year survival rates (often <15%) owing to inherent or acquired treatment resistance, distant recurrence, and limited therapeutic options for late-stage disease (30, 31). These conflicting statistics underscore the urgent unmet need for novel therapeutic strategies to improve long-term outcomes in this aggressive subgroup. In recent years, immune checkpoint inhibitors (ICBs), such as anti-PD-1/PD-L1 antibodies, have revolutionized oncology by reactivating tumor-specific T cell immunity. However, their application in mRC has had limited success. Clinical trials have reported objective response rates (ORRs) below 20% for ICB monotherapy in unselected mRC populations, primarily because of the low mutational burden, defective antigen presentation machinery, and immunosuppressive tumor microenvironment (TME) that characterize many mRC cases (32). Against this backdrop, tumor vaccination, particularly mRNA-based vaccines, has emerged as a promising complementary strategy. By delivering tumor-specific antigens (TSAs) encoded in messenger RNA, these vaccines can stimulate *de novo* T-cell responses against neoantigens, bypassing the need for pre-existing immunity and potentially overcoming ICB resistance (33). However, the clinical translation of mRNA vaccines hinges on two critical challenges: (1) identifying immunogenic, patient-specific TSAs (e.g., those derived from aberrant splicing or frameshift mutations) that can elicit robust T cell activation (34) and (2) understanding the tumor-immune landscape to guide patient selection, as not all mRC subtypes may benefit equally from vaccine-based immunotherapy (35).

Our study directly addresses these gaps through three key contributions. First, we systematically identified FAM135A, a novel neoantigen derived from recurrent aberrant splicing and frameshift mutations, as a potent mRNA vaccine candidate, validated by its high immunogenicity and specificity in silico analyses using retrospective public datasets (TCGA and GEO). Second, we defined two distinct immune subtypes (C1/C2) in mRC, characterized by divergent TME compositions, antigen presentation capacities, and prognostic outcomes, thus providing a framework for precision immunotherapy. Third, we demonstrated that C2 subtype tumors exhibit enhanced responsiveness to ICB, likely due to their enriched CD8+ T cell infiltration and upregulated interferon-γ signaling, thereby establishing a predictive biomarker for combinatorial mRNA vaccine-ICB regimens (**Figure 8**). Collectively, these findings bridge critical gaps in mRC translational research, offering actionable insights into optimizing vaccine design and patient stratification in future clinical trials.

Our study delineated the aberrant transcription patterns and gene mutation profiles of CRC, highlighting the target antigen *FAM135A* as a promising candidate for mRNA vaccine development. Elevated expression of *FAM135A* correlates with poor clinical prognosis and increased infiltration of antigen-presenting cells (APCs) (**Figure 2-J-X**), positioning it as an ideal vaccine target for two mechanistic reasons:(1)high tumor-specific expression generates abundant molecular targets for immune recognition, enabling precise targeting by vaccine-primed immune effectors;(2)The APC-enriched tumor microenvironment potentiates antigen presentation and T-cell priming, actively counteracting potential immune-evasion strategies (e.g., PD-L1-mediated immunosuppression) as previously reported. Thus, therapeutic targeting of *FAM135A* may paradoxically transform its pro-tumorigenic properties into vaccine-induced vulnerabilities, leveraging its dual biological features to enhance immunogenicity while mitigating adaptive resistance mechanisms.

The development of cancer vaccines presents unique challenges (36) that are not encountered with vaccines for infectious diseases. Unlike the latter, preventive cancer vaccines are largely in preclinical stages, with clinical translation hampered by the complexities of antigen prediction and suboptimal immunogenicity of the identified antigens (37). A pivotal hurdle is the identification and delivery of tumor-specific antigens that are both immunogenic and capable of evading immune tolerance mechanisms. Our categorization of CRC into two immune subtypes based on immune gene expression profiles offers a strategic approach for vaccine development. These subtypes, C1 and C2, exhibit distinct molecular and clinical characteristics, with C1 patients showing a more favorable prognosis than C2 patients in both TCGA and GEO databases. Our immune subtyping method demonstrated superior predictive accuracy over existing tumor markers and traditional staging methods, highlighting its potential in prognostic prediction and guiding therapeutic responses to mRNA vaccines.Tumor immune status is a critical determinant of the efficacy of mRNA vaccine. Our analysis revealed that C1 tumors, characterized by a high immune score, may respond more favorably to mRNA vaccines, whereas C2 tumors, with a low immune score, suggest a more immunosuppressive environment that could impede vaccine-induced immune responses. The differential immune cell composition between C1 and C2 subtypes further supports this notion, with C1 exhibiting an “immune-hot” profile and C2 an “immune-cold” phenotype. While our SubMap analysis, utilizing a melanoma cohort, suggested enhanced sensitivity to CTLA-4 and PD-1 blockades in subtype C2, we acknowledge that cross-cancer transcriptomic extrapolation must be interpreted cautiously. To orthogonalize this finding, we assessed intrinsic tumor biomarkers. Subtype C2 demonstrated a significantly higher IFN-γ signature, which strongly correlates with robust intra-tumoral immune activation and favorable ICB outcomes. Intriguingly, classical markers such as MSI status and TMB did not differ significantly between the subtypes. Rectal cancers are typically characterized by a very low incidence of MSI-H (~5-10%), which aligns with our cohort distribution. The elevated IFN-γ signaling and predicted ICB responsiveness in the C2 subtype, despite a predominantly MSS background and lack of TMB elevation, suggest that the robust immune infiltration in these patients may be driven by alternative mechanisms—potentially the presentation of highly immunogenic novel antigens derived from alternative splicing events, such as *FAM135A*. This highlights the value of our classification system in potentially identifying the rare subset of MSS rectal cancer patients who are immunologically “hot” and might benefit from novel immunotherapeutic strategies, though future validation in prospective, ICB-treated rectal cancer cohorts is essential.

Our study is the first to identify RC antigens by screening for aberrant transcripts for mRNA vaccine development. This approach complements previous classifications and provides a novel perspective for vaccine-targeted populations. Interestingly, our findings in melanoma patients, where CTLA-4 and PD-1 inhibitors were effective in the C2 subtype, validated our immune subtyping method. This aligns with other studies that have shown the importance of immune context in determining treatment responses.

## Conclusion

While our study provides a foundation for the development of mRNA vaccines for RC, it is imperative to note that the identified vaccine antigens require further validation. Future research should focus on translating these findings into clinical trials, assessing the safety and efficacy of mRNA vaccines targeting *FAM135A*, and exploring the synergistic effects of combination therapies with immune checkpoint blockade (ICB). Identification of immune subtypes offers a personalized approach to RC treatment, potentially enhancing the efficacy of mRNA vaccines and other immunotherapies. Despite the promising findings regarding the immunogenic potential of *FAM135A* and the robust immune stratification, several limitations of this study must be acknowledged. First, our results are primarily based on comprehensive in silico analyses using retrospective public datasets (TCGA and GEO). Although we applied rigorous statistical filters and multi-dimensional validation algorithms to minimize false positives, the specific immunogenicity and anti-tumor efficacy of *FAM135A*-derived peptides have not yet been validated in wet-lab experiments, such as *in vitro* T-cell activation assays or *in vivo* tumor-bearing mouse models. Therefore, the biological function of *FAM135A* in rectal cancer and its potential as a vaccine target warrant further experimental verification in future studies. Second, the retrospective nature of the cohorts may introduce selection bias, and prospective clinical trials are needed to confirm the predictive value of the immune subtypes.

## Data Availability

The datasets used and/or analyzed during the current study are available from the corresponding author upon reasonable request, and some of the datasets are provided in the **Supplementary Material**.

## Supplementary material

Supplementary Figure 1 Identification and functional analysis of potential antigen candidate genes (A) Venn diagram of the intersection of genes upregulated in AS events and genes with frameshift mutations. (B-D) bubble charts of GO BP, MF, and CC gene set enrichment analysis of potential antigen candidate genes.GO, Gene Ontology; BP, Biological Process; MF, Molecular Function; CC, Cellular Component.

Supplementary Figure 2 Validation of immune subtypes robustness using Non-negative Matrix Factorization (NMF). A. NMF consensus matrix heatmap for TCGA-READ samples at the optimal rank (k = 2). The sharp boundaries and high contrast of the diagonal blocks indicate high intra-cluster stability and clear separation between the two subtypes (C1: n = 109; C2: n = 57). B. Principal component analysis (PCA) plot based on the NMF-derived clustering. Samples are colored by their subtype assignment (C1 in blue; C2 in orange). The confidence ellipses demonstrate a clear spatial separation between the C1 and C2 subtypes, confirming the distinct biological features and the robustness of the stratification. Abbreviations: NMF, non-negative matrix factorization; PCA, principal component analysis; PC1/2, principal component 1/2.

Supplementary Figure 3 Distribution of TCGA-READ molecular subtypes and TNM staging A. Distribution of pathological T staging (T1+T2 vs. T3+T4) in the two subtypes; B. Distribution of pathological N staging (N0 vs. N1+N2) across the two subtypes; C. Distribution of pathological M staging (M0 vs. M1) across the two subtypes; D. Distribution of AJCC overall staging (Stage I–II vs. Stage III–IV) across the two subtypes.

Supplementary Figure 4 Identification of Co-Expression of Immune Genes in TCGA-READ. (A) Sample Clustering in TCGA-READ; (B) Scale-Free Fitting Index as a Function of Soft Threshold Parameter Variation; (C) Average Connectivity of the Network as a Function of Soft Threshold Parameter Variation; (D) Dendrogram of Hierarchical Clustering for Differentially Expressed Genes; (E) Number of Genes in Each Module; F. Differential Distribution of Eigenvectors for Each Module in TCGA-READ Subtypes; G. Univariate Cox Analysis of Module Eigenvectors; (H-J) Bubble Plot of GO Enrichment Results for Prognostically Relevant Modules.
