# Supplementary figures and images for: Comprehensive profiling of alternative splicing and immune landscapes in rectal cancer: implications for mRNA vaccine design and immune subtype stratification
Source: Front Oncol. 2026 Apr 13;16:1780631. doi: 10.3389/fonc.2026.1780631 (PMC13110952; doi:10.3389/fonc.2026.1780631)

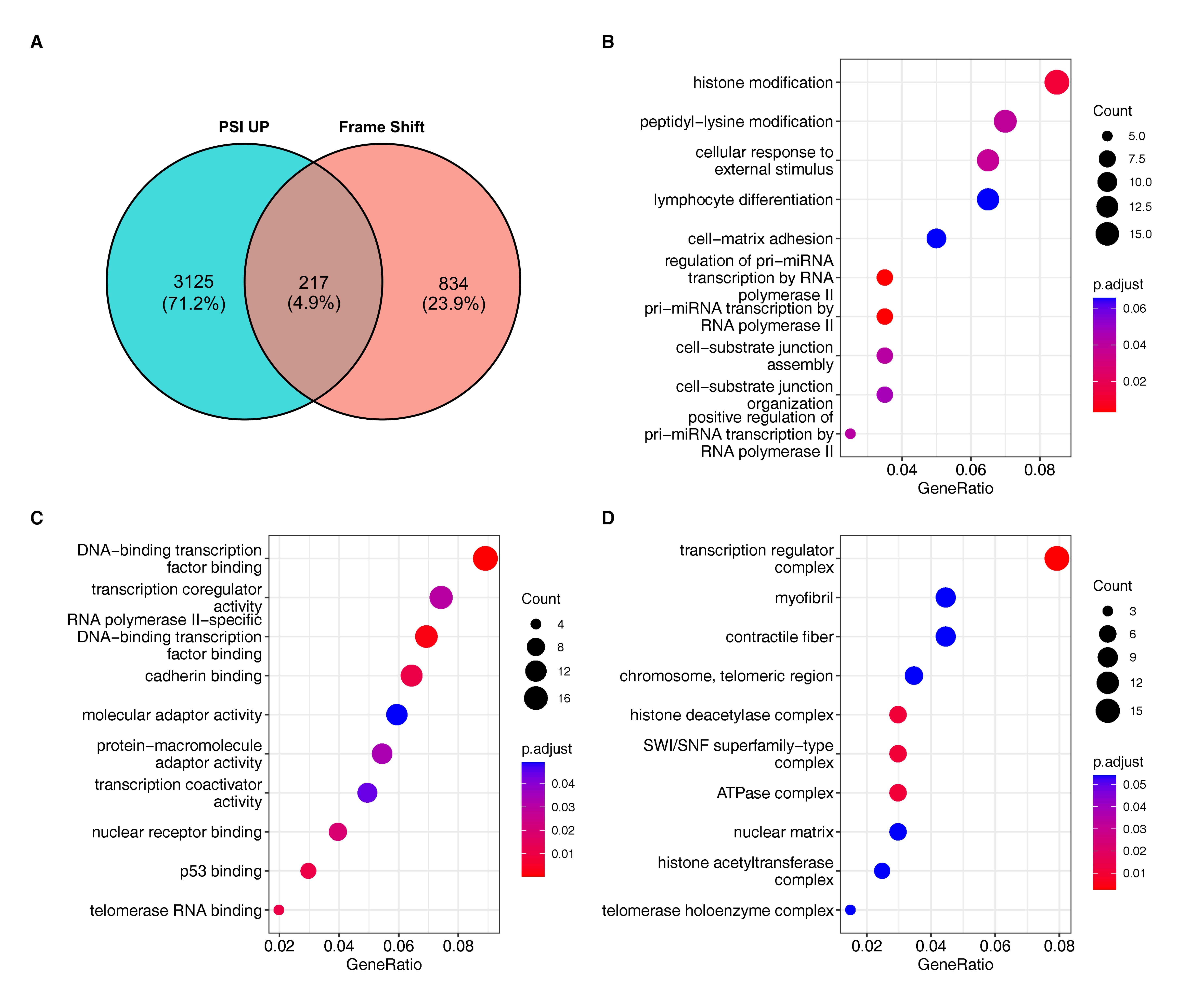

Supplement: Supplementary Figure 1 — Identification and functional analysis of potential antigen candidate genes (A) Venn diagram of the intersection of genes upregulated in AS events and genes with frameshift mutations. (B-D) bubble charts of GO BP, MF, and CC gene set enrichment analysis of potential antigen candidate genes.GO, Gene Ontology; BP, Biological Process; MF, Molecular Function; CC, Cellular Component. [file Image1.jpeg]

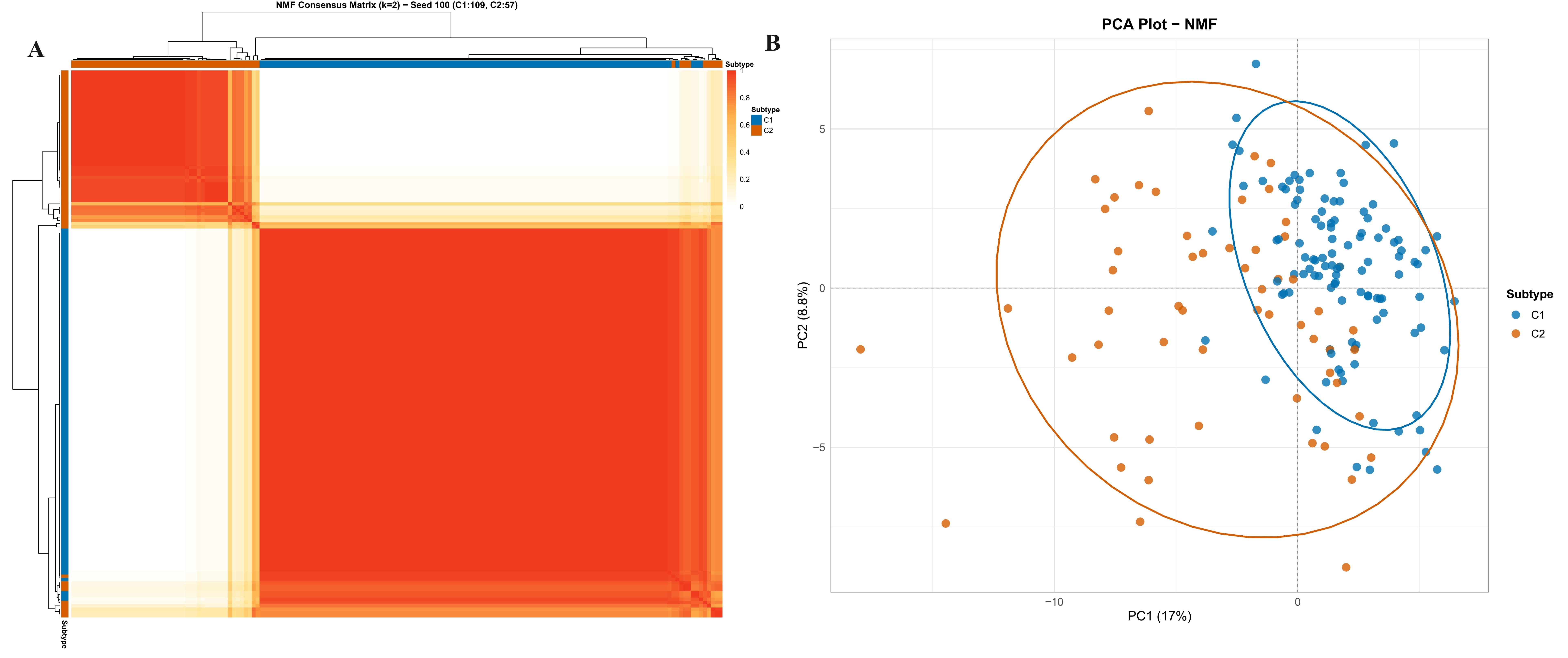

Supplement: Supplementary Figure 2 — Validation of immune subtypes robustness using Non-negative Matrix Factorization (NMF). A. NMF consensus matrix heatmap for TCGA-READ samples at the optimal rank (k = 2). The sharp boundaries and high contrast of the diagonal blocks indicate high intra-cluster stability and clear separation between the two subtypes (C1: n = 109; C2: n = 57). B. Principal component analysis (PCA) plot based on the NMF-derived clustering. Samples are colored by their subtype assignment (C1 in blue; C2 in orange). The confidence ellipses demonstrate a clear spatial separation between the C1 and C2 subtypes, confirming the distinct biological features and the robustness of the stratification. Abbreviations: NMF, non-negative matrix factorization; PCA, principal component analysis; PC1/2, principal component 1/2. [file Image2.jpeg]

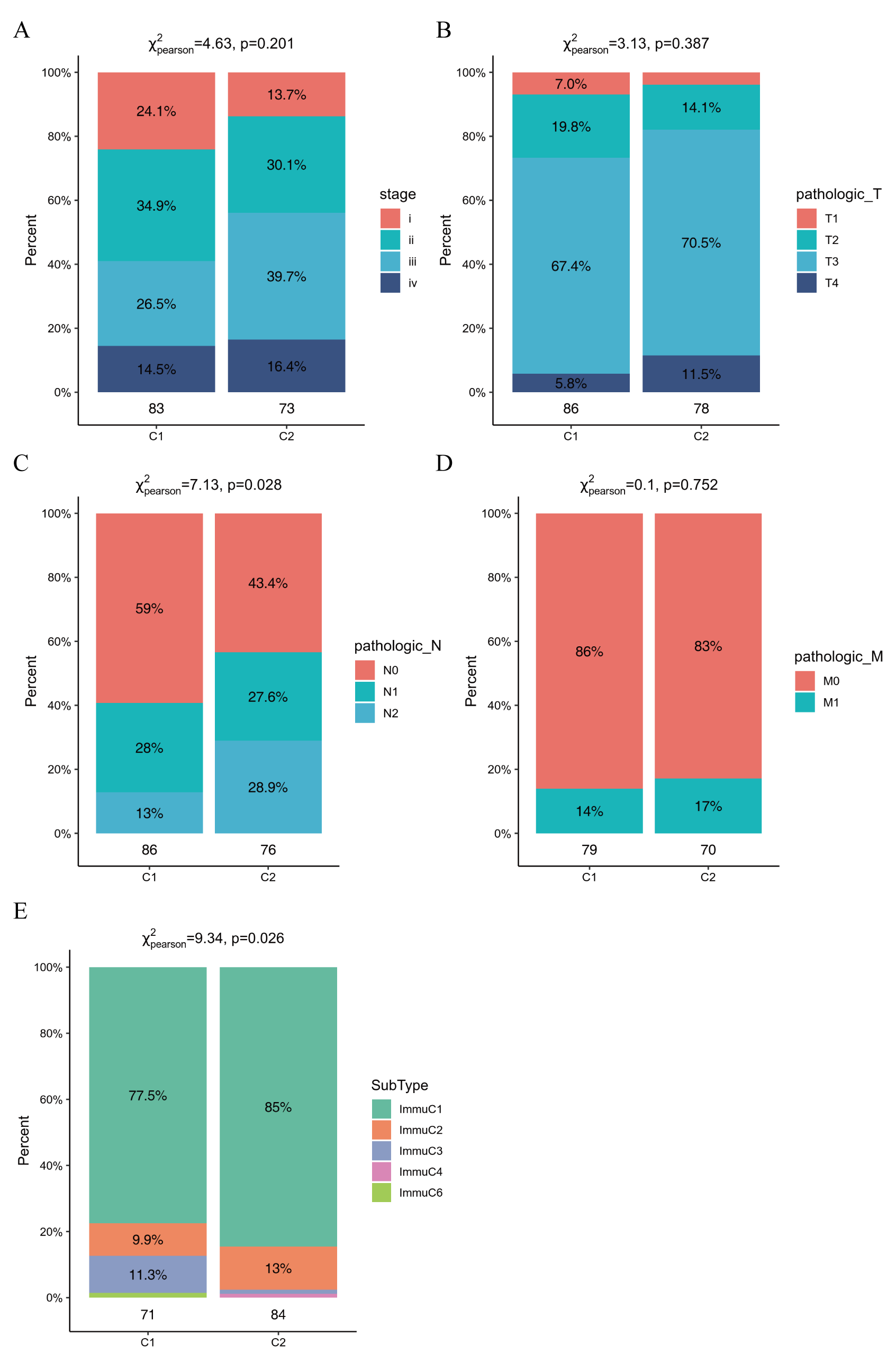

Supplement: Supplementary Figure 3 — Distribution of TCGA-READ molecular subtypes and TNM staging A. Distribution of pathological T staging (T1+T2 vs. T3+T4) in the two subtypes; B. Distribution of pathological N staging (N0 vs. N1+N2) across the two subtypes; C. Distribution of pathological M staging (M0 vs. M1) across the two subtypes; D. Distribution of AJCC overall staging (Stage I–II vs. Stage III–IV) across the two subtypes. [file Image3.tiff]

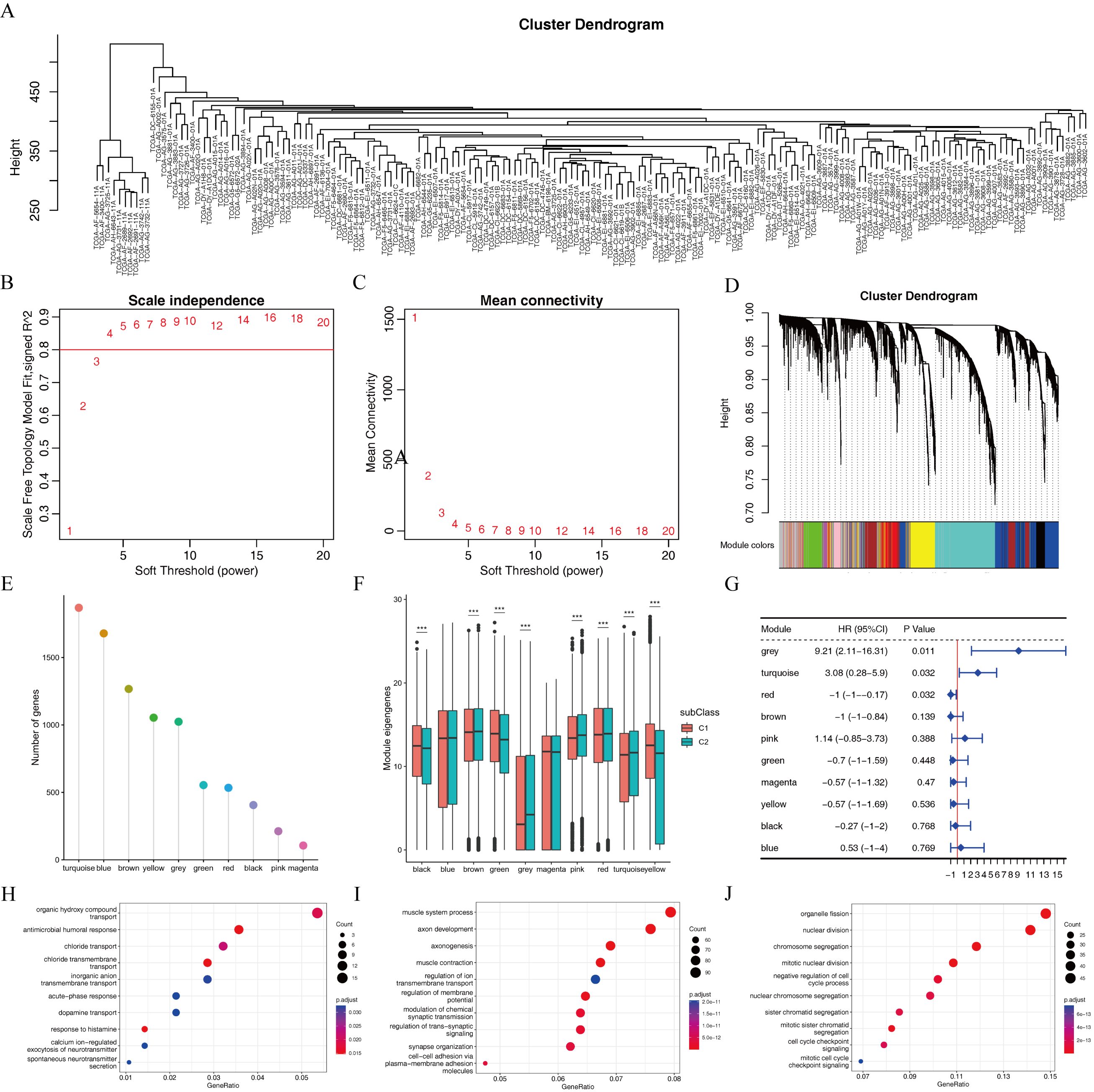

Supplement: Supplementary Figure 4 — Identification of Co-Expression of Immune Genes in TCGA-READ. (A) Sample Clustering in TCGA-READ; (B) Scale-Free Fitting Index as a Function of Soft Threshold Parameter Variation; (C) Average Connectivity of the Network as a Function of Soft Threshold Parameter Variation; (D) Dendrogram of Hierarchical Clustering for Differentially Expressed Genes; (E) Number of Genes in Each Module; F. Differential Distribution of Eigenvectors for Each Module in TCGA-READ Subtypes; G. Univariate Cox Analysis of Module Eigenvectors; (H-J) Bubble Plot of GO Enrichment Results for Prognostically Relevant Modules. [file Image4.tiff]
