# Supplementary material for: Comprehensive profiling of alternative splicing and immune landscapes in rectal cancer: implications for mRNA vaccine design and immune subtype stratification
Source: Front Oncol. 2026 Apr 13;16:1780631. doi: 10.3389/fonc.2026.1780631 (PMC13110952; doi:10.3389/fonc.2026.1780631)
Supplement: Supplementary file 6 [file DataSheet1.pdf]

```
setwd("C:/Users/fjhua/Desktop")

#install.packages("UpSetR")

library(UpSetR)

library(ggplot2)

rt <- readRDS("PSI_df.Rds")

gene=rt[,1]

asType=rt[,3]

upsetList=list(AA=unique(gene[asType=="AA"]),

               AD=unique(gene[asType=="AD"]),

               AP=unique(gene[asType=="AP"]),

               AT=unique(gene[asType=="AT"]),

               ES=unique(gene[asType=="ES"]),

               ME=unique(gene[asType=="ME"]),

               RI=unique(gene[asType=="RI"]) )

upsetData=fromList(upsetList)


pdf(file="upset.pdf",onefile = FALSE,width=9,height=6)

upset(upsetData,

      nsets = 7,

      nintersects = 30,

      order.by = "freq",

      show.numbers = "yes",

      number.angles = 0,

      point.size = 1.5,

      matrix.color= "black",

      line.size = 0.8,

      mainbar.y.label = "Gene Intersections",
```

```
        main.bar.color="#FF0018",
        sets.bar.color="#00CED1",
        sets.x.label = "Set Size",
        sets = sort(names(upsetList), decreasing = TRUE),
        keep.order = T)
dev.off()
```
